# Supplementary material for: The genome sequence of the red-crested pochard, Netta rufina (Pallas, 1773)
Source: Wellcome Open Res. 2024 Oct 21;9:617. [Version 1] doi: 10.12688/wellcomeopenres.23204.1 (PMC11561386; doi:10.12688/wellcomeopenres.23204.1)
Supplement: Peer review history

# Reviewer report: Reviewer response for version 1

**DOI:** 10.21956/wellcomeopenres.25556.r108536

**Published:** 2024-11-13

**Authors:** Leif Andersson

**Affiliations:** 1 Medical Biochemistry and Microbiology, Uppsala University, Uppsala, Sweden

This brief report describes a high-quality genome sequence of the red-crested pochard, a duck with a wide-spread distribution in Eurasia. This will a valuable resource for future research on this species. The data are well presented and I have only minor comments on the paper:

 Background, 1st para. Is there really a minute size difference between males and females, or is this a chance difference noted in a small sample set? A 20 gram difference appears too small to be reported unless it is based on a large dataset. If this is uncertain, it would be better to just write the same maximum weight for both sexes.

 Background, 3rd para: Typo: The incubation period is …

 Background, 4th para: “is found throughout Europe”, this statement is not correct. It does not breed in Iceland, Norway, Finland or Sweden”.

 Table 1. It would be useful to indicate in Table 1 that only muscle tissue was used for RNAseq.

 Table 3. The scaffolds have been numbered according to size. I think it would be better to compare these scaffolds to the chicken genome and use the same chromosome numbering as in chicken because of the high degree of conserved synteny among birds. This will simplify future comparative genomics studies of this species.

Are sufficient details of methods and materials provided to allow replication by others?

Yes

Is the rationale for creating the dataset(s) clearly described?

Yes

Are the datasets clearly presented in a useable and accessible format?

Yes

Are the protocols appropriate and is the work technically sound?

Yes

Reviewer Expertise:

Evolutionary genomics

I confirm that I have read this submission and believe that I have an appropriate level of expertise to confirm that it is of an acceptable scientific standard.

# Reviewer report: Reviewer response for version 1

**DOI:** 10.21956/wellcomeopenres.25556.r108543

**Published:** 2024-11-11

**Authors:** Chen Siang Ng

**Affiliations:** 1 National Tsing Hua University, Hsinchu, Taiwan

This manuscript presents a high-quality genome assembly of the red-crested pochard ( *Netta rufina*), a species of ecological and conservation interest. The authors utilize advanced sequencing methods and provide comprehensive assembly metrics, making this genomic resource valuable for future research on avian genetics, population dynamics, and conservation efforts. The study is well-executed, with rigorous methodology and transparent reporting of results, making a meaningful contribution to avian genomics.

 This manuscript is a valuable addition to avian genomics and conservation biology. It will be well-suited for indexing with minor revisions, especially regarding clarity of objectives, discussion of limitations, and emphasis on the broader significance. The study has clear scientific merit, and the genomic resource presented here has the potential to support impactful research in multiple areas.

 1) Briefly mentioning the genome’s significance or potential applications would strengthen the abstract and better convey the study's relevance.

 2) While the introduction provides a comprehensive background, it would benefit from explicitly stated research objectives at the end, which would help guide readers more effectively.

 3) Some background information in the introduction could be streamlined to focus more directly on framing the research question.

 4) Some recent duck genomes have been published. Please discuss and compare your results:

Are sufficient details of methods and materials provided to allow replication by others?

Yes

Is the rationale for creating the dataset(s) clearly described?

Yes

Are the datasets clearly presented in a useable and accessible format?

Yes

Are the protocols appropriate and is the work technically sound?

Yes

Reviewer Expertise:

Evolutionary genomics, evolutionary ornithology, evo-devo

I confirm that I have read this submission and believe that I have an appropriate level of expertise to confirm that it is of an acceptable scientific standard.

## References

[1] : A high-quality genome and comparison of short- versus long-read transcriptome of the palaearctic duck Aythya fuligula (tufted duck). *Gigascience* .2021;10(12) : 10.1093/gigascience/giab081 10.1093/gigascience/giab081 34927191 PMC8685854

[2] : A new duck genome reveals conserved and convergently evolved chromosome architectures of birds and mammals. *Gigascience* .2021;10(1) : 10.1093/gigascience/giaa142 10.1093/gigascience/giaa142 33406261 PMC7787181

[3] : Genome Assembly and Evolutionary Analysis of the Mandarin Duck Aix galericulata Reveal Strong Genome Conservation among Ducks. *Genome Biol Evol* .2022;14(6) : 10.1093/gbe/evac083 10.1093/gbe/evac083 35640266 PMC9189614

[4] : Chromosome-level genome assembly of the critically endangered Baer's pochard (Aythya baeri). *Sci Data* .2023;10(1) : 10.1038/s41597-023-02063-9 176 10.1038/s41597-023-02063-9 36991020 PMC10060409

[5] : A high-quality chromosomal-level genome assembly of Greater Scaup (Aythya marila). *Sci Data* .2023;10(1) : 10.1038/s41597-023-02142-x 254 10.1038/s41597-023-02142-x 37142629 PMC10160052

[6] : A new chromosome-scale duck genome shows a major histocompatibility complex with several expanded multigene families. *BMC Biol* .2024;22(1) : 10.1186/s12915-024-01817-0 31 10.1186/s12915-024-01817-0 38317190 PMC10845735

[7] : Chromosome-level genome provides insights into evolution and diving adaptability in the vulnerable common pochard (Aythya ferina). *BMC Genomics* .2024;25(1) : 10.1186/s12864-024-10846-6 927 10.1186/s12864-024-10846-6 39363174 PMC11451245
